# Safety awareness, attitudes, and behaviors among university students in Saudi Arabia: implications for pre-employment safety readiness

**DOI:** 10.3389/fmed.2026.1940131

**Published:** 2026-10-05

**Authors:** Mohammed Alsubaie, Sadeq Abu-Dawas, Aiman Alwadi, Sereen Kazim, Narmeen Shaikh, Fouad Jabri, Mohammad Hasan Rajab, Noara Alhusseini, Majed Ramadan

**Affiliations:** 1 Biostatistics, Epidemiology and Public Health, College of Medicine, Alfaisal University, Riyadh, Saudi Arabia; 2 College of Pharmacy, Alfaisal University, Riyadh, Saudi Arabia; 3 University of Jeddah, Jeddah, Saudi Arabia; 4 Department of Global Public Health, Karolinska Institutet, Stockholm, Sweden; 5 Population Health Research Section, King Abdullah International Medical Research Center (KAIMRC), Riyadh, Saudi Arabia; 6 King Saud Bin Abdulaziz University for Health Sciences, Riyadh, Saudi Arabia; 7 Ministry of National Guard—Health Affairs, Riyadh, Saudi Arabia

**Keywords:** occupational health, safety attitudes, safety awareness, safety behaviors, safety culture, Saudi Arabia, university students

## Abstract

**Background:**

Safety culture supports safe learning environments and is widely regarded as a determinant of safety practice, yet evidence among university students in Saudi Arabia remains limited. Because students represent the future workforce in safety-sensitive fields such as healthcare, engineering, and science, characterizing their safety awareness, attitudes, and behaviors is relevant to occupational health practice. This study assessed these three domains among students at a single private university in Riyadh and identified demographic factors associated with them.

**Methods:**

A single-center cross-sectional survey was conducted among a convenience sample of 370 undergraduate and graduate students at Alfaisal University, Riyadh, Saudi Arabia, using a previously validated 19-item questionnaire assessing safety awareness and attitudes (12 items) and safety behaviors (7 items). Descriptive statistics and multivariable generalized linear model (GLM) analyses identified demographic factors independently associated with each domain. Findings are discussed in relation to pre-employment safety readiness; workplace safety behavior and performance were not measured.

**Results:**

Most participants were aged 18–22 years (76.2%), female (62.2%), and undergraduate (86.2%). Mean scores were 3.44 ± 0.71 for safety awareness and attitudes and 3.63 ± 0.69 for safety behavior. Female students scored significantly higher than male students on both safety awareness and attitudes (*β* = 0.27, 95% CI: 0.12 to 0.42; *p* = 0.0006) and safety behavior (*β* = 0.23, 95% CI: 0.08 to 0.38; *p* = 0.002). Non-Saudi students scored significantly higher than Saudi students on both safety awareness and attitudes (*β* = 0.27, 95% CI: 0.10 to 0.44; *p* = 0.001) and safety behavior (*β* = 0.19, 95% CI: 0.03 to 0.36; *p* = 0.010). Adjusted models accounted for a small proportion of variance (R^2^ = 0.082 and 0.070, respectively). Age, academic level, marital status, and college affiliation were not significantly associated with either outcome.

**Conclusions:**

University students showed generally positive yet moderate safety awareness, attitudes, and behaviors. Gender and nationality were the only variables that reached statistical significance in the adjusted models, and the associated effects were small. Because subsequent workplace safety readiness and performance were not measured, these findings describe students at one point in time and do not demonstrate future occupational behavior. They identify pre-workforce groups with lower current scores and provide baseline, hypothesis-generating evidence for multicenter and longitudinal studies in Saudi Arabia.

## 1 Introduction

Safety culture refers to the shared values, beliefs, and underlying assumptions that shape how individuals and organizations perceive and manage risk. Where safety culture is well developed, hazards are more consistently recognized, established procedures more reliably followed, and preventive action taken before incidents occur. These relationships have been described across healthcare, industry, transportation, and education (1, 2), and the available evidence is broadly consistent: a substantial proportion of preventable accidents and adverse events has been attributed to deficits in safety knowledge, limited regulatory compliance, inadequate training, or insufficient organizational prioritization of safety (3, 4). Strengthening safety culture has therefore become a strategic priority for organizations seeking to reduce preventable harm.

The terms safety culture and safety climate are frequently used interchangeably but are conceptually distinct, and the distinction matters for interpreting what the present study measured. Safety culture denotes the relatively enduring, shared values, beliefs, and underlying assumptions regarding safety held within a group or organization. Safety climate refers to the more transient, directly measurable perceptions of the prevailing safety environment at a given point in time, and is commonly characterized as a surface manifestation, or snapshot, of the underlying culture (5, 6). The boundary between the two constructs remains actively debated, with some authors treating climate as a constituent element of culture and others treating culture as the condition under which a climate emerges (5).

Within this framework, three individual-level components are conventionally distinguished. Safety awareness is the cognitive recognition of hazards and of the controls that govern them. Safety attitudes are the affective evaluation of safety and of one's own responsibility for it. Safety behavior is the self-reported or observed enactment of safe practice (7, 8). Awareness, attitudes, and behavior are properties of individuals; safety culture and safety climate are properties of collectives.

This distinction defines the scope of the present study. We measured the three individual-level constructs by self-report among students. We did not assess organizational-level safety culture or safety climate: no institutional artefacts, management-commitment indicators, or aggregated unit-level perceptions were collected, and no multilevel modelling was performed. The term safety culture is therefore used in this paper only as an overarching label for the three measured individual-level domains, and should not be read as a claim to have measured the institutional culture of Alfaisal University.

Interest in the concept expanded following a series of major industrial and technological accidents that demonstrated its importance in high-reliability organizations (9). Organizations with stronger safety cultures have subsequently been reported to exhibit more favorable safety climates, closer adherence to protocols, and lower accident rates (10, 11), and safety-culture assessment has become an established means by which institutions benchmark performance and target improvement (12). This literature has, however, remained concentrated in occupational and industrial settings. Educational institutions, in which students, faculty, and staff encounter physical, environmental, laboratory, and behavioral hazards, have received comparatively little attention, and the academic laboratory studies that do exist have documented substantial variation in perceived safety climate between institutions and user groups (13).

Universities present a distinctive setting. Safety culture in higher education extends beyond accident prevention to emergency preparedness, hazard awareness, and the routine management of a large and heterogeneous campus population. Students encounter laboratory hazards, fire risk, campus security concerns, and general health risks. Surveys of university students in China and Bangladesh have reported only moderate levels of safety awareness, attitudes, and behavior, indicating a gap in safety education at this level (14, 15). A subsequent and considerably larger multi-institution survey of 1,676 students across 16 Bangladeshi universities and medical colleges confirmed this pattern, reporting moderate levels across all safety culture components and identifying age, gender, and institution type as correlates (16). Perrin et al. advanced a comparable argument within chemical engineering education, contending that safety practices established during university persist into professional careers (17), and a recent survey of engineering students assessed against National Examination Board in Occupational Safety and Health topics reported variation in students' understanding of core safety topics across engineering faculties and academic years (18).

University students constitute the incoming workforce in healthcare, engineering, business, law, science, and public service. It is frequently argued that the safety attitudes and habits formed during higher education persist into employment, and early exposure to safety expectations has been linked to regulatory compliance, risk perception, and adherence to workplace procedures among young workers. This proposition is, however, largely inferential. To our knowledge, no longitudinal study has followed a student cohort from university into employment to establish whether student-level safety attitudes predict subsequent workplace safety behavior, and the present cross-sectional study cannot supply that evidence. The university years are therefore treated here as a plausible, but not a demonstrated, point of intervention, and the findings reported below should be read as a description of students rather than as a forecast of the workers they will become.

In Saudi Arabia, safety culture has been examined extensively in healthcare organizations and in high-risk industries such as oil and gas, construction, and manufacturing (19–25). Al Sulaie and Park reported that management commitment and worker attitudes were principal determinants of safety culture among oil and gas employees (21), and Alrehaili documented associations between safety culture, safety behavior, and compliance among construction workers on large government projects (22). Despite this expanding occupational literature, pre-employment safety readiness among university students, the population from which the workforce in these same high-risk sectors is drawn, has not to our knowledge been characterized. The existing evidence describes individuals already in employment and provides no information on the safety knowledge, attitudes, or gaps with which they entered it.

This gap is of direct policy relevance. Saudi Vision 2030 establishes a national agenda for improved quality of education and for human capability development (26). Within its National Transformation Program, the Ministry of Human Resources and Social Development launched the National Strategic Program for Occupational Safety and Health, which rests on four pillars, the first of which is raising awareness of the importance of occupational safety and health and strengthening preventive education (27). That program subsequently produced the National Council for Occupational Safety and Health, established in 2022 as the Kingdom's principal authority in this field (28). Preventive education directed at individuals who have not yet entered the labor market falls within the scope of that first pillar. Characterizing how students currently understand and practice safety therefore generates evidence relevant both to university policy and to national workforce-readiness planning.

This study aimed to assess safety awareness, attitudes, and behavior among students at Alfaisal University, Riyadh, and to identify the demographic factors associated with them. We hypothesized that these measures would vary by gender, nationality, age, academic level, and college affiliation.

Because no baseline data existed for this population, a cross-sectional design was appropriate for establishing current status and generating hypotheses, notwithstanding its inability to support causal inference. The results are intended to inform university safety programs and, more broadly, workforce-readiness planning under Saudi Vision 2030 (26).

## 2 Materials and methods

### 2.1 Study design and sample size

A cross-sectional survey assessed safety culture among students at Alfaisal University in Riyadh, following the STROBE guidelines for cross-sectional studies. Data was collected between March and April 2023 through a self-administered questionnaire distributed via Google Forms; the same approach used in other contemporaneous surveys of Alfaisal University students (29).

Eligible participants were undergraduate and graduate students enrolled in any of the six colleges at Alfaisal University: Business Administration, Engineering, Medicine, Pharmacy, Law and International Relations, and Science and General Studies. The survey link was distributed through university email lists to students across academic levels, genders, and nationalities.

The invitation was circulated to the enrolled student body of approximately 3,100 students. The 370 completed responses therefore correspond to approximately 11.9% of the enrolled student population. Because the survey link was open and could be forwarded, the number of students who received or opened the invitation cannot be established; this figure should therefore be interpreted as a coverage proportion of the enrolled population rather than as a response rate calculated on a verified denominator of invited students. No information was collected on students who did not respond, and respondents and non-respondents could not be compared on any characteristic.

Because no prior safety-culture data existed for this population, the cross-sectional design was chosen specifically to establish that baseline and to identify which demographic factors are associated with it; information that can then guide more targeted interventions.

The minimum sample size was calculated using Cochran's formula with a finite population correction (enrolled student population ≈ 3,100; 95% confidence level; 5% margin of error; 50% response distribution), yielding n_0_ = 384 and a finite-population-corrected target of 342 participants. A total of 370 complete responses was obtained.

This calculation should be interpreted as a precision target rather than as evidence of representativeness. Cochran's formula presupposes probability sampling; recruitment here was by convenience. The calculation therefore establishes the margin of error that would apply to a simple random sample of this size drawn from a population of 3,100, and confirms that the achieved sample is large enough to meet that precision standard, but it does not establish that the achieved sample is representative of the enrolled population. Any departure from representativeness introduced by self-selection is a bias in the point estimates and is not corrected by sample size.

Statistical power for the multivariable models was evaluated *post hoc*. With 370 participants and 11 estimated covariate parameters, corresponding to approximately 34 participants per parameter, the study had 80% power at *α* = 0.05 to detect an overall model effect size of *f*^2^ = 0.047 (equivalent to *R*^2^ ≈ 0.045), and 80% power to detect a single-coefficient effect of *f*^2^ = 0.021. The observed model effect sizes, *f*^2^ = 0.089 for Safety Awareness and Attitudes and *f*^2^ = 0.075 for Safety Behavior, correspond to achieved power of 0.99 and 0.97 respectively. The study was therefore adequately powered for the principal comparisons, but underpowered to detect small differences in sparsely populated subgroups, notably the College of Pharmacy (*n* = 13) and the College of Law and International Relations (*n* = 2).

Recruitment relied on convenience sampling, which is efficient for electronic distribution but introduces the potential for selection bias. To mitigate this, recruitment was directed across all six colleges and all academic levels rather than to any single group, which also ensured the inclusion of disciplines—among them medicine, engineering, and pharmacy—in which safety competence carries directly into subsequent professional practice.

### 2.2 Questionnaire design

The questionnaire was adapted from the safety culture instrument developed by Gong (14) and subsequently applied by Hasan and Younos (15) among university students. It was administered in English, the primary language of instruction at Alfaisal University. The complete instrument, including all 19 items and the demographic section, is provided as Supplementary Material S1.

It has two sections. The first collects demographic information: age, gender, nationality, marital status, college, and academic level. The second measures safety culture across 19 items split into two domains: Safety Awareness and Attitudes (12 items) and Safety Behaviors (7 items).

The instrument treats safety culture as cognitive, affective, and behavioral: the awareness-and-attitude items capture what students know and believe about safety, while the behavior items capture what they actually do.

Responses used a five-point Likert scale from 1 (strongly disagree) to 5 (strongly agree); higher scores reflect more positive safety awareness, attitudes, and behavior.

### 2.3 Validity and reliability

The instrument was adapted from previously validated safety-culture questionnaires. The original validation work reported satisfactory construct, convergent, and discriminant validity together with strong internal consistency; item wording and content were therefore left unchanged in order to preserve those psychometric properties.

This questionnaire has previously demonstrated excellent internal consistency in the studies from which it was adapted: reported Cronbach's alpha coefficients were 0.903 for the Safety Awareness and Attitudes domain and 0.879 for the Safety Behavior domain, with an overall coefficient of 0.870 (14, 15).

Internal consistency was also calculated directly on the present sample (*N* = 370). Cronbach's alpha was 0.855 for the Safety Awareness and Attitudes domain, 0.746 for the Safety Behavior domain, and 0.895 for the full 19-item instrument. These sample-specific values indicate good to acceptable internal consistency, somewhat lower than reported in the original validation studies but within the range generally considered adequate for research use.

Validation in the present sample was limited to internal consistency. No exploratory or confirmatory factor analysis was performed on these data, and no formal cross-cultural adaptation procedure—expert content review, cognitive interviewing, or pilot testing within a Saudi student population—was undertaken prior to administration. The two-domain structure was therefore assumed from the source instruments (14, 15) rather than demonstrated in this population. Cronbach's alpha establishes that the items within each assumed domain covary; it does not establish that the assumed two-factor structure is the correct one for Saudi students, nor that the items carry equivalent meaning in this context. Construct validity in the Saudi setting consequently remains unestablished, and this is acknowledged in the Limitations.

### 2.4 Statistical analysis

Data were exported from Google Forms and analyzed using SAS statistical software, version 9.4 (SAS Institute Inc., Cary, NC, USA). Prior to analysis, data were examined for completeness, consistency, and accuracy. All submitted questionnaires were complete, and no missing data was identified.

Descriptive statistics summarized participant characteristics and safety culture scores: continuous variables as means, medians, and standard deviations, and categorical variables as frequencies and percentages. Unadjusted differences in composite domain scores between demographic groups (Figures 1, 2) are presented descriptively and were not subjected to formal significance testing; all inferential comparisons were conducted within the multivariable models described below. Normality of the composite scores and homogeneity of variance were assessed prior to modelling, and residual diagnostics are reported in Section 2.4. All tests were two-tailed, with significance set at *α* = 0.05.

**Figure 1:**
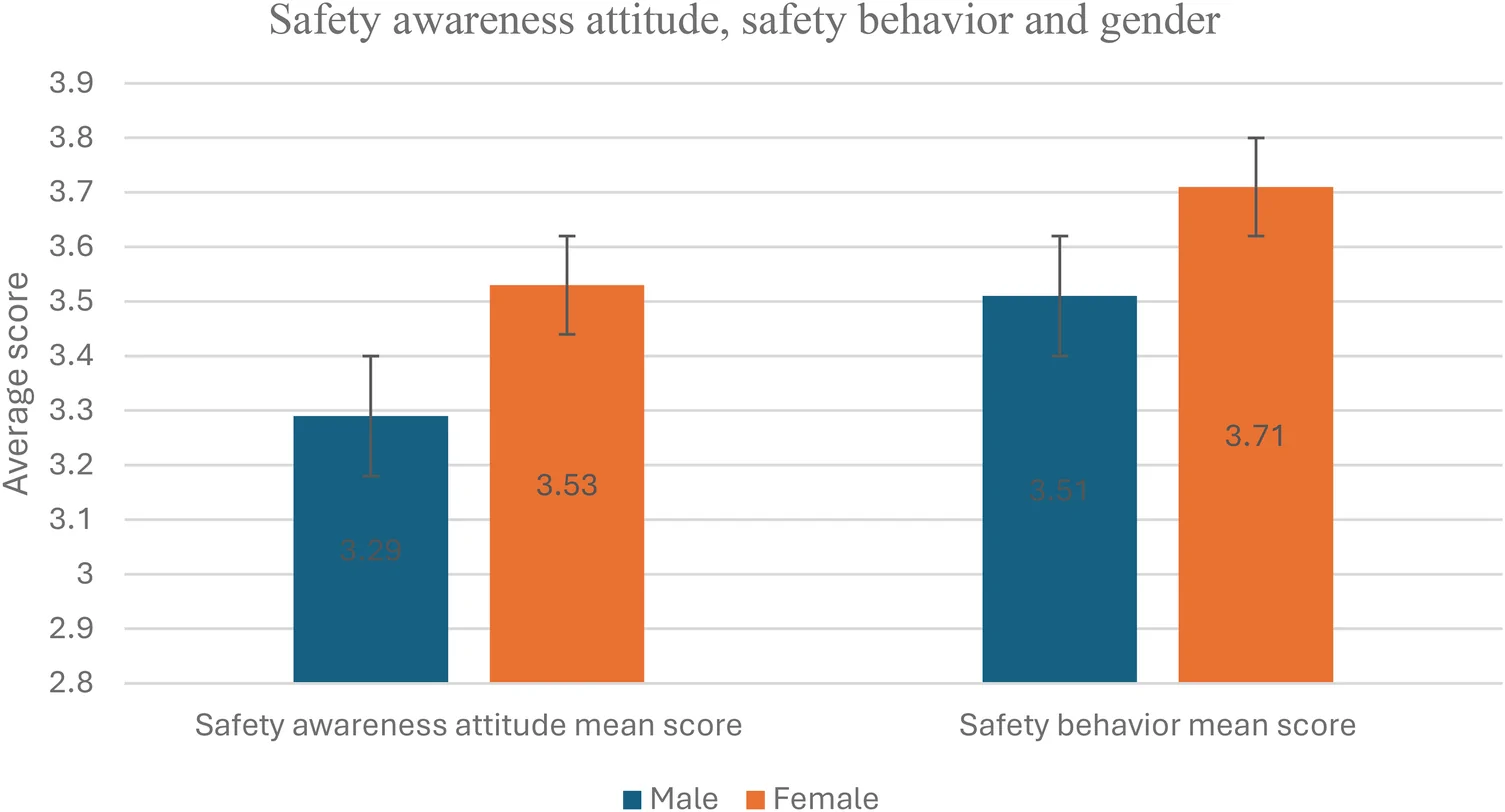
Mean safety awareness and attitudes and safety behavior scores by gender.

**Figure 2:**
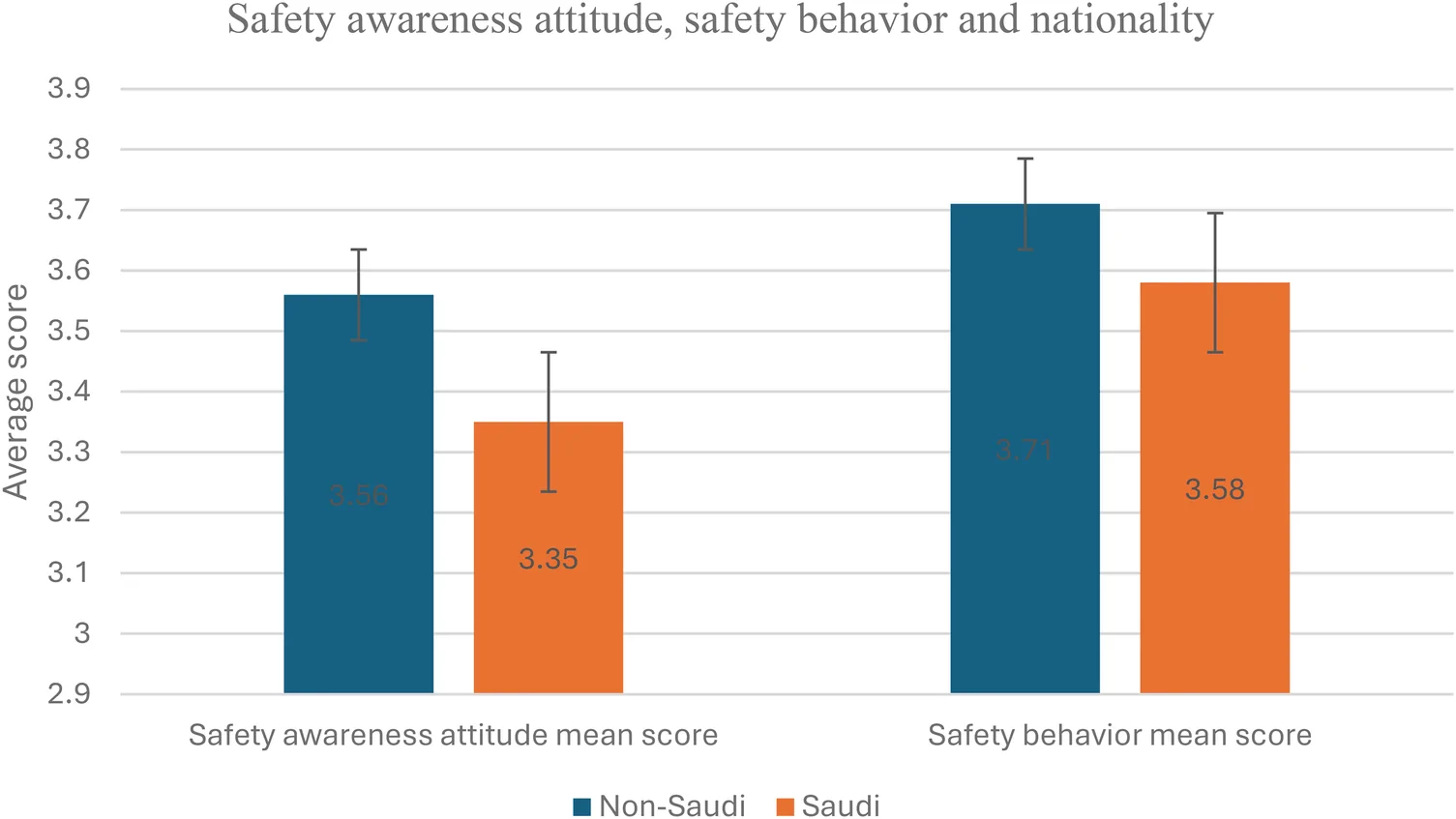
Mean safety awareness and attitudes and safety behavior scores by nationality.

Composite scores were calculated as the mean of the constituent items for the Safety Awareness and Attitudes domain and for the Safety Behavior domain, giving both domains a possible range of 1 to 5, and were treated as continuous variables consistent with previous safety culture research. Higher scores reflected greater safety awareness, more positive safety attitudes, and safer behaviors. No items required reverse-coding: an independent check of item-total correlations using the response-level data confirmed that all 19 items, including the three items with negatively-worded stems (for example, “I never used forbidden high-powered electric equipment”), loaded in a consistent positive direction.

For interpretive purposes only, composite scores were categorized as low (1.00–2.33), moderate (2.34–3.67), or high (3.68–5.00). These bands divide the 1–5 response range into three equal intervals of 1.33 scale points, calculated as (5 − 1)/3, a convention widely applied to five-point Likert composites. The cut-points were therefore specified *a priori* by the structure of the response scale rather than derived from the observed distribution of the data, and no distributional or empirical criterion was used to place them. The categorization served only to aid interpretation; all inferential analyses were performed on the continuous composite scores, and no result reported in this paper depends on the placement of these cut-points. The observed means, 3.44 and 3.63, both fall within the moderate band.

For multivariable analysis, a generalized linear model (GLM) with a Gaussian distribution and an identity link function, statistically equivalent to multivariable linear regression, was used to identify demographic factors independently associated with Safety Awareness and Attitudes scores and with Safety Behavior scores. Independent variables comprised age, gender, nationality, college affiliation, academic level, and marital status. Regression coefficients (*β*), 95% confidence intervals, and corresponding *p*-values are reported for every coefficient. For categorical variables with more than two levels, namely age group and college affiliation, Type III omnibus *F* tests were computed to assess the overall contribution of the variable to the model, in addition to the individual contrasts against the reference category.

All statistical tests were two-tailed, with significance set at *p* < 0.05, and model fit was assessed using *R*^2^ and adjusted *R*^2^. Model assumptions were checked directly against the response-level data. Residuals were normally distributed for both outcomes (Jarque–Bera and Kolmogorov–Smirnov tests, both *p* > 0.5), and variance was reasonably constant across fitted values (Breusch–Pagan test on all covariates: *p* = 0.167 for Safety Awareness and Attitudes, *p* = 0.598 for Safety Behavior).

Variance inflation factors were below 5 for every predictor except the 18–22-year age group and, to a lesser extent, the College of Engineering and College of Medicine indicators, for which VIFs were approximately 7 to 9. The explanation is structural: 99.6% of participants aged 18–22 years were undergraduates, so age group and academic level convey substantially overlapping information in this sample.

A sensitivity analysis was therefore conducted in which academic level was removed from both models. Removing academic level changed the gender and nationality coefficients by less than 0.001 in absolute value in both domains, leaving the direction, magnitude, and statistical significance of both associations unchanged. Collinearity therefore does not materially affect the study's principal conclusions, although the age-group coefficients specifically have inflated variance and should be interpreted with corresponding caution (30).

Standardized effect sizes were calculated for the statistically significant associations as Cohen's d, defined as the unadjusted difference in mean domain score between the two groups divided by the pooled standard deviation of that domain. Cohen's d was therefore computed from the observed group means rather than from the adjusted regression coefficients, and is reported alongside, not in place of, the adjusted *β* and its confidence interval. Values of 0.20, 0.50, and 0.80 were interpreted as small, medium, and large respectively.

### 2.5 Ethical considerations

The study was conducted in accordance with the ethical principles outlined in the Declaration of Helsinki. Ethical approval was obtained from the Institutional Review Board of Alfaisal University (Approval No. IRB-20203) prior to data collection.

Participation was voluntary, and all responses were collected anonymously. An information sheet explaining the study objectives, procedures, and participants' rights was provided at the beginning of the survey; submitting a completed questionnaire was taken as informed consent.

Because this study examines safety awareness, attitudes, and behavior among individuals preparing to enter safety-critical professions, the responsible use of its findings warrants explicit consideration. The intent of the work is to inform the design of student safety training rather than to characterize any group as deficient.

## 3 Results

### 3.1 Participant characteristics

A total of 370 students completed the survey and were included in the analysis. Most respondents were aged 18–22 years (76.2%), followed by those aged 23–27 years (11.4%), 28–31 years (8.4%), and 32 years or older (4.1%). Most participants were female (62.2%), undergraduate (86.2%), unmarried (94.6%), and Saudi nationals (58.4%). Every college at Alfaisal University was represented, with the largest proportions enrolled in Medicine (39.2%) and Engineering (38.4%) (Table 1).

**Table 1:** Demographic characteristics of the participants (alfaisal university students, *N* = 370).

Characteristic	*N* (%)^1^
Age	
18–22 years	282 (76.22)
23–27 years	42 (11.35)
28–31 years	31 (8.38)
32 years and above	15 (4.05)
Gender	
Male	140 (37.84)
Female	230 (62.16)
College	
College of Business Administration	51 (13.78)
College of Engineering	142 (38.38)
College of Science and General Studies	17 (4.59)
College of Medicine	145 (39.19)
College of Pharmacy	13 (3.51)
College of Law and International Relations	2 (0.54)
Academic level	
Graduate (Master's)	51 (13.78)
Undergraduate	319 (86.22)
Marital status	
Married	20 (5.41)
Unmarried	350 (94.59)
Nationality	
Saudi	216 (58.38)
Non-Saudi	154 (41.62)
Safety awareness and attitudes score (mean/median/SD)	**3.44/3.50/0.71**
Safety behavior score (mean/median/SD)	**3.63/3.71/0.69**

1 N, sample size; %, percentage. Scoring: strongly agree = 5, agree = 4, neither agree nor disagree = 3, disagree = 2, strongly disagree = 1. Marital status was dichotomized as married vs. unmarried; the unmarried category comprises single (*n* = 349) and divorced (*n* = 1) respondents.

Bold values indicates (mean/median/SD): Mean, Median, Standard Deviation.

### 3.2 Safety culture scores

Overall, participants showed moderate levels of safety culture. The mean score for the Safety Awareness and Attitudes domain was 3.44 (SD = 0.71; median = 3.50), and for the Safety Behavior domain, 3.63 (SD = 0.69; median = 3.71) (Table 1). Students generally hold positive views about safety and act on them reasonably often, but there is clear room to strengthen both further.

Gender and nationality both showed up as descriptive differences before any adjustment. Female students scored higher than male students on both Safety Awareness and Attitudes (3.53 vs. 3.29) and Safety Behavior (3.71 vs. 3.51) (Figure 1). Non-Saudi students scored higher than Saudi students on both Safety Awareness and Attitudes (3.56 vs. 3.35) and Safety Behavior (3.71 vs. 3.58) (Figure 2).

### 3.3 Factors independently associated with safety culture

Multivariable analyses identified demographic factors independently associated with Safety Awareness and Attitudes scores and Safety Behavior scores, adjusting for age, gender, nationality, marital status, academic level, and college affiliation (Table 2).

**Table 2:** Association between demographic factors and safety awareness/attitudes and safety behavior scores among Alfaisal university students (generalized linear model).

	Safety awareness & attitudes		Safety behavior	
	Estimate (*β*) [95% CI]	*p*-value	Estimate (β) [95% CI]	*p*-value
Age		*Type III p* *=* 0*.*45		*Type III p* *=* 0*.*17
18–22 years	−0.31 [−0.79 to 0.17]	0.21	−0.44 [−0.91 to 0.03]	0.07
23–27 years	−0.16 [−0.60 to 0.28]	0.48	−0.25 [−0.70 to 0.20]	0.28
28–31 years	−0.11 [−0.57 to 0.35]	0.64	−0.17 [−0.60 to 0.26]	0.44
32 years and above (ref.)	—	—	—	—
Gender				
Male (ref.)	—	—	—	—
Female	**0.27 [0.12 to 0.42]**	**0** **.** **0006** *	**0.23 [0.08 to 0.38]**	**0** **.** **002** *
College		*Type III p* *=* 0*.*48		*Type III p* *=* 0*.*74
College of Business Administration	−0.24 [−0.65 to 0.17]	0.25	−0.03 [−0.34 to 0.28]	0.85
College of Engineering	−0.08 [−0.41 to 0.25]	0.64	0.08 [−0.25 to 0.41]	0.64
College of Science and General Studies	0.08 [−0.39 to 0.55]	0.74	0.09 [−0.34 to 0.52]	0.68
College of Medicine	−0.12 [−0.48 to 0.24]	0.51	0.11 [−0.26 to 0.48]	0.56
Pharmacy/Law & International Relations (ref.)	—	—	—	—
Academic level				
Graduate (Master's)	−0.01 [−0.21 to 0.19]	0.92	−0.02 [−0.33 to 0.29]	0.90
Undergraduate (ref.)	—	—	—	—
Marital status				
Married	0.18 [−0.13 to 0.49]	0.25	0.12 [−0.25 to 0.49]	0.52
Unmarried (ref.)	—	—	—	—
Nationality				
Saudi (ref.)	—	—	—	—
Non-Saudi	**0.27 [0.10 to 0.44]**	**0** **.** **001** *	**0.19 [0.03 to 0.36]**	**0** **.** **010** *

* *p* < 0.05. Model: generalized linear model with Gaussian distribution and identity link (equivalent to multivariable linear regression), adjusted for all variables shown. ref. = reference group. β = regression coefficient; CI = confidence interval. Model fit: Safety Awareness and Attitudes, R^2^ = 0.082 (adjusted R^2^ = 0.054); Safety Behavior, R^2^ = 0.070 (adjusted R^2^ = 0.041), calculated directly from the response-level data. Pharmacy College (*n* = 13) and the College of Law and International Relations (*n* = 2) were combined into a single reference category for the college-affiliation covariate because each had too few respondents to support a stable, separately estimated coefficient. Type III omnibus tests assess the overall contribution of age group and college affiliation, each of which has more than two levels.

The labels of the table explains what each of the bold values are that are provided in Table 2.

For Safety Awareness and Attitudes, female students scored significantly higher than male students (*β* = 0.27, 95% CI: 0.12 to 0.42; *p* = 0.0006), and non-Saudi students scored significantly higher than Saudi students (*β* = 0.27, 95% CI: 0.10 to 0.44; *p* = 0.001). Age group, marital status, academic level, and college affiliation were not significantly associated with this domain, and the Type III omnibus tests for age group and college affiliation were likewise non-significant (*p* = 0.45 and *p* = 0.48, respectively).

The same pattern was observed for Safety Behavior: female students scored significantly higher than male students (*β* = 0.23, 95% CI: 0.08 to 0.38; *p* = 0.002), and non-Saudi students scored significantly higher than Saudi students (*β* = 0.19, 95% CI: 0.03 to 0.36; *p* = 0.010), with no significant associations for age group, marital status, academic level, or college affiliation, and non-significant Type III omnibus tests for age group (*p* = 0.17) and college affiliation (*p* = 0.74).

Gender and nationality were the only variables that reached statistical significance in the adjusted models, and they did so consistently across both domains: female and non-Saudi students scored higher than male and Saudi students respectively, after adjustment for the remaining demographic characteristics (Table 2; Figure 3). Non-significance for the other variables should not be interpreted as evidence that no association exists, particularly for the sparsely populated college categories, where the study was underpowered to detect differences of the size observed for gender and nationality.

**Figure 3:**
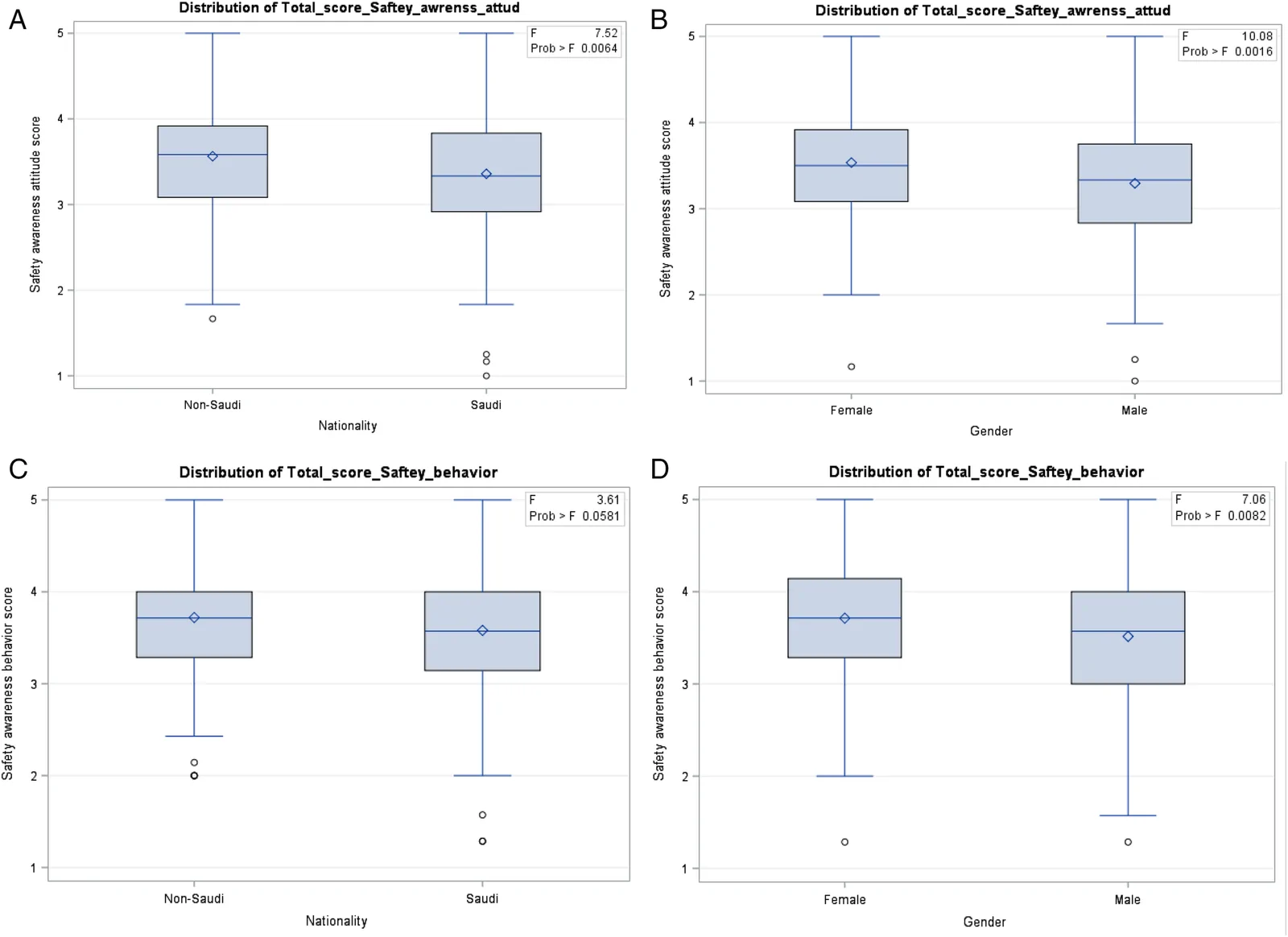
Distribution of safety awareness and attitudes and safety behavior composite scores by nationality and gender. **(A)** Safety Awareness and Attitudes score by nationality. **(B)** Safety Awareness and Attitudes score by gender. **(C)** Safety Behavior score by nationality. **(D)** Safety Behavior score by gender.

Ninety-five percent confidence intervals and standardized effect sizes were calculated for the four statistically significant associations. For Safety Awareness and Attitudes, the female-vs.-male difference was *β* = 0.268 (95% CI: 0.116 to 0.420; Cohen's d = 0.34) and the non-Saudi-vs.-Saudi difference was *β* = 0.269 (95% CI: 0.104 to 0.435; d = 0.30). For Safety Behavior, the corresponding values were *β* = 0.233 (95% CI: 0.083 to 0.383; d = 0.29) for gender and *β* = 0.195 (95% CI: 0.031 to 0.358; d = 0.19) for nationality. None of the four confidence intervals included zero. By conventional benchmarks these represent small effects. The associations are statistically robust but account for only a small proportion of the variance in domain scores, with the full models explaining 8.2% and 7.0% of variance respectively.

## 4 Discussion

This study provides baseline data on safety awareness, attitudes, and behavior among university students in Saudi Arabia. Participants showed moderate levels of safety awareness and attitudes (mean = 3.44) and safety behavior (mean = 3.63), indicating an established but incomplete foundation. Gender and nationality were the only variables that reached statistical significance in the adjusted models, with female and non-Saudi students scoring higher across both domains, although the associated effects were small. Because these students will enter healthcare, engineering, science, and business, their current safety knowledge and attitudes are of potential relevance beyond the campus; the extent to which they carry into professional practice was not measured here and remains to be established.

As an exploratory cross-sectional study, these results are best read as foundational: a first description of where things stand and which student groups may need more support, rather than a test of cause and effect. That is exactly the kind of evidence needed before designing and evaluating targeted interventions in a population that had no prior safety-culture data at all.

The moderate levels observed here are consistent with Gong's study of Chinese undergraduates and with Hasan and Younos's survey of Bangladeshi university students (14, 15), and with the larger multi-institution Bangladeshi study subsequently reported by Ahsan et al., which found moderate levels across all safety culture components among 1,676 students (16). Students across these settings report reasonably positive safety attitudes without correspondingly consistent safety practice. The convergence of findings across four countries suggests that this gap between attitude and practice is not specific to any single educational system, and points consistently toward the same implication: safety education, laboratory training, emergency-preparedness drills, and campus-wide awareness activities have scope to be strengthened.

The most consistent finding was the difference by gender. Female students scored significantly higher than male students on both domains, a pattern consistent with previous work among university students in China, Bangladesh, Trinidad, and the United States (14, 15, 31, 32), and partially replicated in the recent multi-institution Bangladeshi study, in which female students scored significantly higher on safety perception and awareness and on the third measured domain, although male students scored significantly higher on safety beliefs and values (16); the direction of the gender difference in that study therefore depended on the domain examined. The mechanism underlying this association cannot be determined from the present data. Gender differences in risk perception and regulatory compliance have been reported in occupational settings (33), but the present study measured no psychological, experiential, or educational variables capable of testing that or any alternative explanation. The association may equally reflect unmeasured factors, including differential prior exposure to safety training, differences in the safety content of secondary or university curricula, differences in laboratory or clinical placement experience, or differential response tendency on self-report instruments. The consistency of the association across two independent domains and its replication in other national settings support its robustness; they do not identify its cause.

Non-Saudi students scored higher than Saudi students on both domains. This contrasts with Hasan and Younos, who reported no residency-related differences among Bangladeshi students (15). The mechanism underlying this association is unknown and cannot be established from these data. The present study measured nationality as a binary administrative category and collected no information on country of origin, length of residence in Saudi Arabia, prior schooling system, prior safety training, or language of previous instruction. Any of these unmeasured variables could account for the observed difference, and nationality may function here as a proxy for one or more of them rather than as an explanatory variable in its own right. The non-Saudi group is also internally heterogeneous, comprising students from multiple countries and educational backgrounds who are combined into a single category by the study design; a difference in mean score between such a group and Saudi nationals is therefore difficult to attribute to any specific characteristic. We note this finding as robust and unexplained. Establishing its basis would require data on prior safety education and schooling history, which future studies should collect.

No significant associations were observed for age, academic level, marital status, or college affiliation, broadly consistent with the findings of Hasan and Younos (15). Two explanations are possible and cannot be distinguished with these data. Safety attitudes and behavior in this population may depend more on individual disposition and campus-wide institutional factors than on academic discipline or year of study; alternatively, uniform safety policies and training provision across Alfaisal University's colleges may attenuate differences that would otherwise be detectable. A third possibility is limited statistical power: the college categories in particular contain as few as 2 and 13 respondents, and the study could not have detected differences of the magnitude observed for gender or nationality within them. These findings differ from research among construction workers, in which educational level moderated the relationship between learning ability, safety culture, and safety performance (34), and from a survey of engineering students that found safety-topic understanding to vary by faculty and academic year (18), suggesting that the dynamics of a still-forming student population may differ from those of an established workforce, or that this single institution is unusually homogeneous in its safety provision. Larger multi-institution studies are required to distinguish these possibilities.

Several practical implications follow. Safety education could be incorporated into student orientation and coursework rather than left to occur incidentally; regular awareness activities, emergency drills, and laboratory safety workshops would reinforce it, and student-led safety ambassador programs have been implemented successfully at some institutions. Structured, leadership-driven approaches to developing safety culture, of the kind established in healthcare organizations (35), may offer a template for university administrators. It should be emphasized, however, that gender and nationality together account for only a small proportion of the variance in domain scores, approximately 7 to 8%. The observed pattern is therefore better characterized as campus-wide than as concentrated in particular groups, which indicates that university-wide programming is the more appropriate primary intervention, with additional attention to lower-scoring groups as a secondary refinement rather than the principal strategy.

These findings also raise a governance question that universities have not generally had to address: where institutional responsibility for student safety culture resides. In most institutions it is located with facilities or security functions concerned with physical hazards, rather than with any unit responsible for the safety attitudes and practices students are forming for later professional use. Treating pre-employment safety readiness as an explicit institutional responsibility, with a designated owner, a defined place in the curriculum, and a mechanism for monitoring change over time, would represent a shift from periodic awareness campaigns toward a structured implementation strategy. This proposal is consistent with the first pillar of the Kingdom's National Strategic Program for Occupational Safety and Health, which identifies the raising of safety awareness and the strengthening of preventive education as a national priority (27). Whether such an approach would produce measurable change in student safety behavior, and whether any such change would persist into employment, are empirical questions that this study cannot answer and that would require intervention and longitudinal designs to address. On this evidence, university safety education is best understood as a plausible point of intervention in the preventive chain preceding occupational health programs, rather than a demonstrated one.

### 4.1 Strengths and limitations

This study has several strengths. To our knowledge it is among the first to examine safety awareness, attitudes, and behavior among university students in Saudi Arabia. It employed a previously validated instrument that demonstrated acceptable to good internal consistency in the present sample, and recruited students from every college and academic level at the institution, providing a reasonably broad picture across the university. The study also reports 95% confidence intervals and standardized effect sizes alongside significance tests, and includes a sensitivity analysis for collinearity. The results provide baseline data against which future studies and occupational health programs may benchmark.

Several limitations should be acknowledged. The cross-sectional design cannot establish causality; it was selected to generate baseline evidence in a previously unstudied population, and the associations reported here should be regarded as hypothesis-generating rather than conclusive.

Most importantly, subsequent workplace safety readiness and safety performance were not measured. No participant was followed into employment, no occupational outcome was recorded, and no linkage to workplace safety data was undertaken. The interpretation of these findings in terms of pre-employment safety readiness is therefore conceptual rather than empirical: this study measured what students report about safety while at university, and the relationship between those reports and subsequent occupational behavior is assumed on the basis of prior literature rather than demonstrated here. Establishing that relationship would require a longitudinal design following graduates into the workforce.

Construct validation in the Saudi context is a second principal limitation. As described in Section 2.3, no exploratory or confirmatory factor analysis was conducted on these data and no formal cross-cultural adaptation procedure was undertaken. The two-domain structure was assumed from instruments validated in Chinese and Bangladeshi student populations (14, 15), and its applicability to Saudi students has not been demonstrated. Measurement invariance across the gender and nationality groups compared here was likewise not assessed, which is directly relevant to interpreting the two principal findings: an observed group difference in mean score could in principle arise from differential item functioning rather than from a genuine difference in the underlying construct.

The study was conducted at a single private, English-medium university whose student demographics, resources, and safety infrastructure are likely to differ from those of the public universities that educate the majority of Saudi nationals. These findings should therefore be treated as hypothesis-generating for the private-university context specifically and should not be generalized to the broader Saudi student population until replicated with multicenter data including public institutions. This caution is reinforced by the multi-institution Bangladeshi study, in which students at private institutions reported significantly lower safety perception and awareness than those at public institutions (16), indicating that institution type may itself be associated with student safety culture. Convenience sampling may have introduced selection bias, as students with a pre-existing interest in safety may have been more likely to respond, and self-reported data carry the risk of social desirability and recall bias. Non-response bias cannot be excluded: no data were collected on students who did not participate, and they may differ systematically from those who did.

Internal consistency in this sample (Cronbach's *α* = 0.746 to 0.895, Section 2.3) was somewhat lower than in the original validation studies, particularly for the Safety Behavior domain, which should be considered when weighing that domain's results. Age and academic level were substantially collinear in this sample, with 99.6% of participants aged 18–22 years being undergraduates; this inflates the variance of the age coefficients specifically and, as the sensitivity analysis in Section 2.4 demonstrates, does not affect the gender or nationality estimates, but the age results should be interpreted with caution. Finally, several academic subgroups, notably Pharmacy (*n* = 13) and Law and International Relations (*n* = 2), were represented by very small numbers, so comparisons involving college affiliation should be interpreted cautiously; the study was underpowered to detect real differences in these groups.

## 5 Conclusion

Students at Alfaisal University demonstrated generally positive but moderate safety awareness, attitudes, and behavior, indicating a reasonable foundation with scope for further development. Gender and nationality were the only variables significantly associated with these outcomes in the adjusted models, with female and non-Saudi students scoring consistently higher across both domains, although the effect sizes were small and the mechanisms underlying these associations were not established.

These results indicate several practical directions: integrating safety education more deliberately into orientation, coursework, and campus life for the entire student body rather than targeting male or Saudi students specifically. As discussed above, the observed group differences, while statistically significant, are small in magnitude, and campus-wide programming is therefore likely to represent the more effective investment.

The relevance of these findings extends beyond the campus. These students will enter healthcare, engineering, science, and business, and it is widely held that safety practices formed during higher education persist into professional life. That proposition, however, was not tested in this study and remains, on current evidence, an assumption rather than a demonstrated relationship. Strengthening safety awareness and practice during the university years is therefore best regarded as a plausible investment in later workplace safety and in the effectiveness of the occupational health programs that follow, the value of which should be established by longitudinal and intervention research.

This study represents an initial investigation rather than a definitive account. Multicenter studies across Saudi universities, including public institutions, would establish national benchmarks and determine whether the nationality association observed here is reproducible. Longitudinal research tracking the development of safety awareness and behavior across a student's time at university, and intervention studies evaluating whether targeted programs produce measurable change, are the appropriate next steps. Studies of this kind would align with the objectives of Saudi Vision 2030 and with the preventive education pillar of the National Strategic Program for Occupational Safety and Health (26, 27).

## Data Availability

The raw data supporting the conclusions of this article will be made available by the authors, without undue reservation.
