# Supplementary material for: Safety awareness, attitudes, and behaviors among university students in Saudi Arabia: implications for pre-employment safety readiness
Source: Front Med (Lausanne). 2026 Oct 5;13:1940131. doi: 10.3389/fmed.2026.1940131 (PMC13677514; doi:10.3389/fmed.2026.1940131)
Supplement: Supplementary file 1 [file Supplementaryfile1.docx]

STROBE Statement Checklist of items that should be included in reports of observational studies

|  | Item No | Recommendation | **Reported on page/line (Manuscript)** |
| --- | --- | --- | --- |
| **Title and abstract** | 1 | (*a*) Indicate the study’s design with a commonly used term in the title or the abstract | Abstract, Methods sentence (line 24): “A cross-sectional survey was conducted...”. Design term appears in the Abstract; the Title itself does not include a design term; optional to add for full compliance. |
|  |  | (*b*) Provide in the abstract an informative and balanced summary of what was done and what was found | Abstract (lines 17–44): structured Background/Methods/Results/Conclusions summary with key findings and effect estimates. |
| Introduction | | |  |
| Background/rationale | 2 | Explain the scientific background and rationale for the investigation being reported | Introduction (lines 47–98); explicit knowledge-gap statement (lines 90–94): “...no study has yet examined pre-employment safety readiness among university students...” |
| Objectives | 3 | State specific objectives, including any prespecified hypotheses | Introduction, closing paragraphs (lines 99–101): objectives stated and hypothesized variation by gender, nationality, age, academic level, and college specified. |
| Methods | | |  |
| Study design | 4 | Present key elements of study design early in the paper | Methods §2.1, first sentence (line 110): cross-sectional design and STROBE guideline use stated at the outset. |
| Setting | 5 | Describe the setting, locations, and relevant dates, including periods of recruitment, exposure, follow-up, and data collection | Methods §2.1 (lines 110–113): setting (Alfaisal University, Riyadh), data-collection window (March–April 2023), and mode (Google Forms) described. |
| Participants | 6 | (*a*) *Cohort study*: Give the eligibility criteria, and the sources and methods of selection of participants. Describe methods of follow-up  *Case-control study*: Give the eligibility criteria, and the sources and methods of case ascertainment and control selection. Give the rationale for the choice of cases and controls  *Cross-sectional study*: Give the eligibility criteria, and the sources and methods of selection of participants | Methods §2.1 (lines 114–117): eligibility (enrolled students, all 6 colleges) and recruitment (university email lists) described. |
|  |  | (*b*) *Cohort study*: For matched studies, give matching criteria and number of exposed and unexposed  *Case-control study*: For matched studies, give matching criteria and the number of controls per case | Not applicable. Unmatched cross-sectional convenience sample; no matching was performed. |
| Variables | 7 | Clearly define all outcomes, exposures, predictors, potential confounders, and effect modifiers. Give diagnostic criteria, if applicable | Methods §2.2 (lines 131–142): demographic variables, the 19-item instrument, its two domains, and the 5-point Likert scoring are all defined. |
| Data sources/ measurement | 8* | For each variable of interest, give sources of data and details of methods of assessment (measurement). Describe comparability of assessment methods if there is more than one group | Methods §2.2–2.3 (lines 131–157): instrument source (Gong 2019; Hasan & Younos 2020) and psychometric properties, including sample-specific Cronbach's alpha, described. Single self-administered instrument; comparability across groups not separately assessed since only one measurement method was used. |
| Bias | 9 | Describe any efforts to address potential sources of bias | Methods §2.1 (lines 125–129) and Limitations (lines 384–404): convenience-sampling bias and mitigation (broad recruitment across colleges/levels) discussed; self-report and social-desirability bias, and non-response bias, discussed in Limitations. |
| Study size | 10 | Explain how the study size was arrived at | Methods §2.1 (lines 121–124): Cochran's formula for finite populations; target n=342; achieved n=370. |
| Quantitative variables | 11 | Explain how quantitative variables were handled in the analyses. If applicable, describe which groupings were chosen and why | Methods §2.4 (lines 168–177): composite scores as item means (possible range 1–5); a priori low/moderate/high thresholds (1.00–2.33 / 2.34–3.67 / 3.68–5.00) defined before Results. |
| Statistical methods | 12 | (*a*) Describe all statistical methods, including those used to control for confounding | Methods §2.4 (lines 159–183): chi-square/Fisher's exact test for univariate comparisons; generalized linear model (GLM) for multivariable analysis; confounders adjusted for (age, gender, nationality, college, academic level, marital status) listed explicitly. |
|  |  | (*b*) Describe any methods used to examine subgroups and interactions | NOT REPORTED. No formal subgroup or interaction-term analyses were performed or described. Flagged as a gap; consider adding a sentence noting this was outside scope, or testing key interactions (e.g., gender × nationality) if space allows. |
|  |  | (*c*) Explain how missing data were addressed | Methods §2.4 (lines 161–163): “All submitted questionnaires were complete, and no missing data was identified.” |
|  |  | (*d*) *Cohort study:* If applicable, explain how loss to follow-up was addressed  *Case-control study:* If applicable, explain how matching of cases and controls was addressed  *Cross-sectional study*: If applicable, describe analytical methods taking account of sampling strategy | PARTIAL. Convenience sampling is described (§2.1) but no design-effect or weighting adjustment for the sampling strategy is reported. This is a minor, already-acknowledged limitation of convenience sampling rather than a missing analysis. |
|  |  | (*e*) Describe any sensitivity analyses | Methods §2.4 (lines 184–195): sensitivity checks reported, variance inflation factors for all predictors, and re-estimation with academic level removed to test whether the age/academic-level collinearity affected the gender and nationality findings. |

Continued on next page

| Results | | |  |
| --- | --- | --- | --- |
| Participants | 13* | (a) Report numbers of individuals at each stage of study; eg numbers potentially eligible, examined for eligibility, confirmed eligible, included in the study, completing follow-up, and analysed | NOT REPORTED. The manuscript gives only the final analytic sample size (n=370); the number of students invited/approached, response rate against that denominator, and any flow diagram are not reported. This is a genuine, currently unresolved gap (recruitment-flow data was not available in any source material provided). |
|  |  | (b) Give reasons for non-participation at each stage | NOT REPORTED. Reasons for non-participation are not available; see note above. |
|  |  | (c) Consider use of a flow diagram | NOT USED. No flow diagram is included. Consider adding one if invited/approached numbers become available. |
| Descriptive data | 14* | (a) Give characteristics of study participants (eg demographic, clinical, social) and information on exposures and potential confounders | Table 1 (page with Table 1) and Results §3.1 (lines 208–214): demographic characteristics and safety-culture composite scores reported in full. |
|  |  | (b) Indicate number of participants with missing data for each variable of interest | Methods §2.4 (line 162): “no missing data was identified” applies to all variables, including those in Table 1. |
|  |  | (c) *Cohort study*: Summarise follow-up time (eg, average and total amount) | Not applicable. Cross-sectional design, no follow-up period. |
| Outcome data | 15* | *Cohort study*: Report numbers of outcome events or summary measures over time | Table 1 (composite score means/medians/SDs) and Results §3.2 (lines 215–224): summary measures for both outcome domains, overall and by gender/nationality. |
|  |  | *Case-control study:* Report numbers in each exposure category, or summary measures of exposure | Not applicable (case-control specific). |
|  |  | *Cross-sectional study:* Report numbers of outcome events or summary measures | Satisfied by the cross-sectional row above. |
| Main results | 16 | (*a*) Give unadjusted estimates and, if applicable, confounder-adjusted estimates and their precision (eg, 95% confidence interval). Make clear which confounders were adjusted for and why they were included | Table 2 and Results §3.3 (lines 225–249): unadjusted group means (Figures 1–2) and adjusted GLM estimates (Table 2) both reported; 95% confidence intervals and Cohen's d effect sizes for the two significant predictors added (lines 241–249), with adjustment set stated in the Table 2 footnote. |
|  |  | (*b*) Report category boundaries when continuous variables were categorized | Methods §2.4 (lines 175–177): low/moderate/high composite-score boundaries defined and reported. |
|  |  | (*c*) If relevant, consider translating estimates of relative risk into absolute risk for a meaningful time period | Not applicable, Outcome is a continuous composite score, not a relative-risk/binary outcome. |
| Other analyses | 17 | Report other analyses done; eg analyses of subgroups and interactions, and sensitivity analyses | Methods §2.4 sensitivity analyses (lines 189–195) and Results effect-size paragraph (lines 241–249). |
| Discussion | | |  |
| Key results | 18 | Summarise key results with reference to study objectives | Discussion, opening paragraph (lines 309–316): key results summarized directly against the stated objectives. |
| Limitations | 19 | Discuss limitations of the study, taking into account sources of potential bias or imprecision. Discuss both direction and magnitude of any potential bias | Strengths and Limitations (lines 384–404): cross-sectional design, single private institution, convenience sampling, self-report bias, sample-specific reliability, age/academic-level collinearity, small subgroup sizes, and non-response bias all discussed, including direction of likely bias where relevant. |
| Interpretation | 20 | Give a cautious overall interpretation of results considering objectives, limitations, multiplicity of analyses, results from similar studies, and other relevant evidence | Discussion throughout, e.g., lines 317–320 (results framed as foundational/hypothesis-generating, not causal), lines 358–362 (effect sizes explicitly described as modest, R²≈7–8%), lines 384–392 (limitations-aware interpretation). |
| Generalisability | 21 | Discuss the generalisability (external validity) of the study results | Limitations (lines 387–392): explicit statement that findings are hypothesis-generating for the private-university context specifically and should not be generalized to Saudi Arabia's broader (majority public-university) student population without multicenter replication. |
| Other information | | |  |
| Funding | 22 | Give the source of funding and the role of the funders for the present study and, if applicable, for the original study on which the present article is based | End matter, Funding section: “The author(s) declare that no financial support was received for the research, authorship, and/or publication of this article.” |

*Give information separately for cases and controls in case-control studies and, if applicable, for exposed and unexposed groups in cohort and cross-sectional studies.

**Note:** An Explanation and Elaboration article discusses each checklist item and gives methodological background and published examples of transparent reporting. The STROBE checklist is best used in conjunction with this article (freely available on the Web sites of PLoS Medicine at http://www.plosmedicine.org/, Annals of Internal Medicine at http://www.annals.org/, and Epidemiology at http://www.epidem.com/). Information on the STROBE Initiative is available at www.strobe-statement.org.
