# Supplementary material for: Safety awareness, attitudes, and behaviors among university students in Saudi Arabia: implications for pre-employment safety readiness
Source: Front Med (Lausanne). 2026 Oct 5;13:1940131. doi: 10.3389/fmed.2026.1940131 (PMC13677514; doi:10.3389/fmed.2026.1940131)
Supplement: Supplementary file 2 [file Supplementaryfile2.docx]

**Supplementary Material S1**

Questionnaire

Survey English version:

Dear Participant,

We are inviting you to participate in our study. We are a team from the MPH program at the College of Medicine at Alfaisal University. The main objective of this study is to assess the safety culture of Alfaisal University students. Participation is voluntary, you can withdraw at any time. All answers will be kept confidential. Filling out the survey will be construed as consent. The survey should not take more than 5-10 minutes. Kindly, check the answer category that most describes you. Your time and effort are greatly appreciated.

* Participation is restricted to only Alfaisal University students aged 18 years and above.

For contact: Mohammed Badi Alsubaie

Department: Public Health, College of Medicine, Alfaisal University.

E-mail: [mbalsubaie@alfaisal.edu](mailto:mbalsubaie@alfaisal.edu)

Safety culture:

Demographic data of respondents:

- Age
  - 18 – 22 years
  - 23- 27 years
  - 28 – 31 years
  - 32 years & Above
- Gender
  - Male
  - Female
- Nationality
  - Saudi
  - Non-Saudi
- Marital status
  - Single
  - Married
  - Divorced
  - Widowed
- College:
  - College of Business Administration.
  - College of Engineering.
  - College of Medicine
  - College of Pharmacy.
  - College of Sciences and General Studies.
  - College of Law and International Relations.
- Academic level:
  - Undergraduate.
  - Graduate. (Master).

Safety awareness and attitude:

1. I always pay attention to or consider risks caused by changes in my living and studying environment at the university.
   - Strongly Disagree
   - Disagree
   - Neither Agree nor Disagree
   - Agree
   - Strongly Agree
2. I often consider how to escape and respond in case of emergencies, like earthquakes or fire.
- Strongly Disagree
- Disagree
- Neither Agree nor Disagree
- Agree
- Strongly Agree
3. I always carefully observe the safety instructions when I go out of campus.
- Strongly Disagree
- Disagree
- Neither Agree nor Disagree
- Agree
- Strongly Agree
4. I pay close attention to the safety instructions in the university regulation.
- Strongly Disagree
- Disagree
- Neither Agree nor Disagree
- Agree
- Strongly Agree
5. I always learn from accidents relating to students.
- Strongly Disagree
- Disagree
- Neither Agree nor Disagree
- Agree
- Strongly Agree
6. I pay close attention to accidents relating to students in the public media.
- Strongly Disagree
- Disagree
- Neither Agree nor Disagree
- Agree
- Strongly Agree
7. I would like to participate in the training, publicity campaign, and emergency response exercises organized by my university.
- Strongly Disagree
- Disagree
- Neither Agree nor Disagree
- Agree
- Strongly Agree
8. When I find other students using high-powered and risky electrical equipment, I always try to stop them and politely remind them of the risk.
- Strongly Disagree
- Disagree
- Neither Agree nor Disagree
- Agree
- Strongly Agree
9. I always pay attention to moving vehicles on campus and escape without delay when the vehicle gets close to me.
- Strongly Disagree
- Disagree
- Neither Agree nor Disagree
- Agree
- Strongly Agree
10. I always pay attention to the necessary preparation practice and wear the necessary clothes, when doing sports.
- Strongly Disagree
- Disagree
- Neither Agree nor Disagree
- Agree
- Strongly Agree
11. I always pay attention to safety considerations when I go back home from my university during the holidays.
- Strongly Disagree
- Disagree
- Neither Agree nor Disagree
- Agree
- Strongly Agree
12. I always carefully listen to or read safety instructions when I enter a laboratory or work in it.
- Strongly Disagree
- Disagree
- Neither Agree nor Disagree
- Agree
- Strongly Agree

Safety Behavior:

1. I always follow the safety signs to behave safely on the university campus.
   - Strongly Disagree
   - Disagree
   - Neither Agree nor Disagree
   - Agree
   - Strongly Agree
2. I always enter and exit a building following the indicated directions when there are large-scale activities in buildings on campus.
   - Strongly Disagree
   - Disagree
   - Neither Agree nor Disagree
   - Agree
   - Strongly Agree
3. When I find out hazards with desks or chairs in the classroom, I always remind other students of the hazards and report them to the related authorities.
   - Strongly Disagree
   - Disagree
   - Neither Agree nor Disagree
   - Agree
   - Strongly Agree
4. I always follow the signs in the stairway when I go upstairs or downstairs.
   - Strongly Disagree
   - Disagree
   - Neither Agree nor Disagree
   - Agree
   - Strongly Agree
5. I never used forbidden high-powered electric equipment in my home.
   - Strongly Disagree
   - Disagree
   - Neither Agree nor Disagree
   - Agree
   - Strongly Agree
6. always put the connection panel into a dry place and away from combustibles and water.
   - Strongly Disagree
   - Disagree
   - Neither Agree nor Disagree
   - Agree
   - Strongly Agree
7. I never plug the mobile phone charger into the terminal board for a long time whether I use it or not.
- Strongly Disagree
- Disagree
- Neither Agree nor Disagree
- Agree
- Strongly Agree
